# Temperature-Induced Variations in Slip Behavior of Single Crystal Aluminum: Microstructural Analysis

**DOI:** 10.3390/ma17092084

**Published:** 2024-04-29

**Authors:** Cheng Tang, Dongfeng Shi, Jin Zhang

**Affiliations:** 1 Light Alloy Research Institute, Central South University, Changsha 410083, China; chengtang456@163.com (C.T.); dongfeng.shi@csu.edu.cn (D.S.); 2 State Key Laboratory of Precision Manufacturing for Extreme Service Performance, Central South University, Changsha 410083, China

**Keywords:** aluminum, single crystal, low-temperature deformation, slip band, orientation effects

## Abstract

The simultaneous increase in strength and plasticity of aluminum and its alloys at cryogenic temperatures has been shown in previous research, but the deformation mechanism was still unclear. Therefore, the purpose of this investigation was to reveal the relationship between slip behavior and mechanical response at low temperatures. A quasi-in situ scanning electron microscope was used to observe the evolution of slip bands in the selected aluminum single crystals with two typical orientations at 25 °C, −100 °C, and −180 °C. The results showed that irrespective of orientation, the density of the slip plane was increased with the decline in temperature, which inhibited slip localization and significantly improved plasticity and work hardening. In detail, at RT, the slip bands were widening until the micro-cracks were generated, causing early failure during deformation. When the temperature was decreased to −180 °C, the slip plane density was increased, and the deformation was more homogenous. Moreover, the slip mode was influenced by orientation and temperature. In particular, a single slip system was activated in the sample with the [112] orientation at all the temperatures investigated. Multiple slip systems were found to activate at 25 °C and −100 °C, and only the primary slip system was activated in the sample with [114] orientation at −180 °C. These findings deepen the understanding of slip behavior at cryogenic temperatures, providing new insights into the deformation mechanism of aluminum and its alloys.

## 1. Introduction

Due to its low density, high strength, and exceptional damage tolerance, aluminum and its alloys have garnered a lot of attention. This makes them a desirable option for the aerospace and automotive industries, which fabricate thin-walled components to decrease weight [1,2,3]. Due to its limited plasticity at room temperature, aluminum alloy components may fracture, consequently restricting their application [4]. Numerous studies have demonstrated that low temperatures may greatly increase the strain hardening, plasticity, and strength of aluminum alloys [5,6,7,8]. Aluminum and its alloys experience plastic deformation at room temperature and low temperatures primarily due to the slip of dislocations along crystallographic planes. Aluminum and its alloys have the four most close-packed planes of different orientations ([111] planes), and each plane has three most close-packed directions (<110> direction), so aluminum has 12 equivalent slip systems. Dislocation slips and the mechanical characteristics of Al alloy are closely linked; for example, strength is determined by the difficulty of dislocation movement, while plasticity requires easy dislocation motion [9,10]. The work hardening of aluminum alloy is mainly affected by the behavior of dislocation—namely, activation of slip systems, dislocation storage, or recovery [11]. At the same time, the mechanical response is also closely related to the slip mode. Cross-slip, for example, can eliminate stress and thus reduce dislocation accumulation. If the back stress generated by the dislocation accumulation is strong enough, the stress peak can be relaxed by local flow on the secondary slip system [12]. At low temperatures, the cross-slip of aluminum and its alloys is inhibited [13,14], resulting in higher dislocation density and work hardening [14]. In addition, according to Taylor’s strain hardening theory [15], the multi-slip process results in stronger dislocation interaction due to higher overall dislocation density. The dynamics of dislocation motion are related to deformation temperature [16], so at low temperatures, the aluminum alloy exhibits different slip behavior and mechanical properties than at room temperature. A detailed study of the different processes that determine the dislocation motion, including dislocation nucleation, dislocation annihilation, and dislocation interactions, etc., is necessary to comprehend the Al alloy’s cryogenic deformation behavior. After the plastic strain of the sample, slip bands will occur on the surface of the polished metal. After imaging the slip band morphology, it can be associated with the long-term dislocation motion during deformation.

Glazer et al. [17] discovered that at RT deformation, there are a lot of straight dislocations generated by plane slip, which is a manifestation of the localization of slip; reducing the temperature promotes the creation of abundant dislocation tangles. Coarse slip bands were seen to occur during RT deformation, according to Yuan et al. [18]. At cryogenic, the slip behavior changes from single to multiple, and the slip band becomes finer, which suppresses the formation of localization slip occurring at low temperatures. Gruber et al. [14] discovered the restraint of cross-slip at low temperatures by in-situ transmission electron microscopy, which led to a high density of screw dislocation at cryogenic. They attributed the increased ductility at cryogenic of the aluminum alloy to an excellent work hardening rate and a very homogeneous deformation distribution due to the reduced number of slip bands. Xu et al. [5] observed that the slip band is wider due to slip localization being present at 295 K than at 77 K for Al–Mg–Si alloy. They attributed the homogeneous strain distribution at low temperatures to dispersed slip. Although a lot of work has been conducted on the dislocation slip of aluminum alloy during low-temperature deformation, the low-temperature plastic deformation of aluminum and its alloys is a complex process, which is related to many factors, including grain size, crystallographic orientation, grain boundaries, solute atoms, etc., and the coupling effects of these factors will cause complex effects on the slip behavior of low-temperature dislocation. There are still many controversies in the study of dislocation behavior at low temperatures, such as the slip band density after deformation at low temperatures and RT, the transformation of slip mold at low temperatures, and its causes. Compared with polycrystalline materials, pure aluminum single crystal has the characteristics of a single composition, uniform orientation, and no grain boundary interference, and the plastic deformation process is easy to repeat under certain conditions. Exploring materials with simple microstructure, such as single crystal materials, will be the beginning of studying the relationship between the macroscopic deformation behavior of materials and the microscopic deformation mechanism of the foundation. As mentioned above, although a lot of investigations tried to explain the origin of simultaneous enhancement in both strength and plastic in Al and its alloy, the deformation mechanism was still unclear.

The purpose of this research is to address the cause of the simultaneous enhancement in both the strength and plastic of aluminum under cryogenic. Therefore, this study quantitatively investigates the impact of the temperatures on the deformation mechanism in the two selected Al single crystals by EBSD-assisted slip trace analysis at 25 °C, −100 °C, and −180 °C. This work presents a novel viewpoint on the source of the simultaneous increase in strength and ductility in aluminum at low temperatures, in addition to updated design principles for low-temperature workability and deformation behavior.

## 2. Materials and Methods

**Figure 1:**
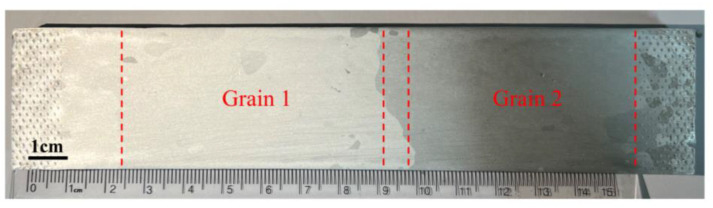
The macro-grain morphology of pure Al after cyclic 0.7% pre-stretching deformation and 560 °C high-temperature annealing.

**Figure 2:**
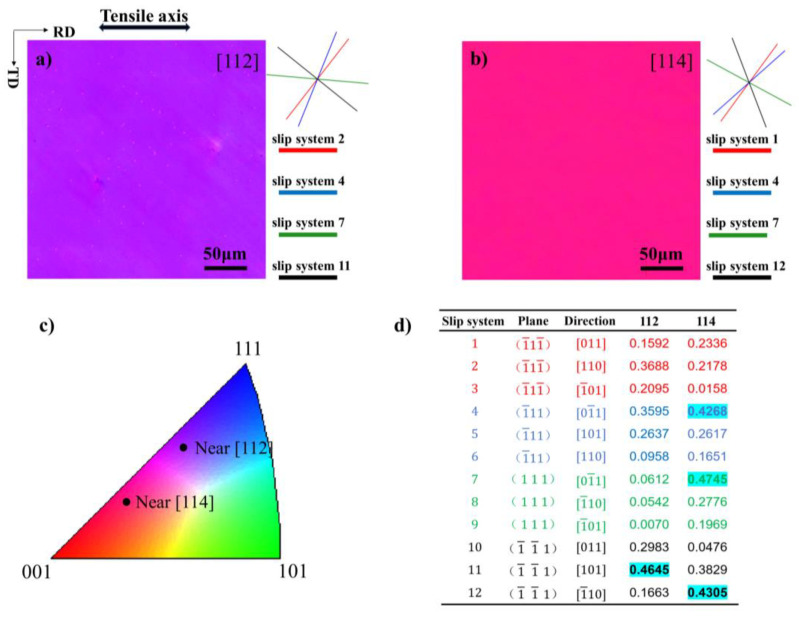
EBSD IPF maps of single crystal aluminum, on the right-hand, 4 potential slip bands and the slip system with the highest Schmid factor (SF) in each slip plane: (**a**) the [112] orientation; (**b**) the [114] orientation; (**c**) stereographic triangle representing the orientation; (**d**) the SF of every slip system.

The 80 mm-thick 99.99% pure Al ingot was cold-rolled to 12 mm thickness over 6 passes, each with a 27% reduction. Next, macro-grain Al was grown by cyclic 0.7% pre-stretching deformation and 560 °C high-temperature annealing [19]. Figure 1 shows the macro-grain morphology of pure Al after cyclic 0.7% pre-stretching deformation and 560 °C high-temperature annealing, and the maximum grain size is about 6.7 cm. The single crystal samples with [112] and [114] orientations were chosen by electron backscattered diffraction (EBSD), as shown in Figure 2a,b.

The slip band and microstructure of two orientations were studied by Scanning electron microscope (SEM)and EBSD utilizing a field emission gun microscope (ZEISS, Jena, Germany) EVO M10. Using a beam current of 10 nA and an accelerating voltage of 20 V, 0.6 μm step size EBSD data gathering was carried out. Diamond pastes with increasingly smaller particles were used to mechanically mirror-polish SEM and EBSD samples. The samples were then completed with a slurry of colloidal silica.

Tensile samples of single crystals of aluminum with a width of 3 mm, thickness of 2.5 mm, and 10 mm in gage length were electro-discharge-machined from a macro-grain Al strip. A CMT5105GL type tensile tester with a low-temperature chamber at 25 °C, −100 °C, and −180 °C was used to conduct tensile testing, with an initial strain rate of 10^−3^/s. At strains of around 5, 10, 15, and 20%, uniaxial tensile tests were terminated to observations of slip band development by SEM.

Slip bands are the straight lines that form when an active slip plane intersects with the surface of a sample, in order to determine the activity of various slip systems, slip band analysis by EBSD-assisted could be applied. First, the MATLAB code [20] took the Euler angles of [112] and [114] orientations of single crystals as input and generated a representation of the 12 slip systems; four probable slip planes were presented on the right of Figure 2a,b. Additionally, the code provided Schmid factors, as illustrated in Figure 2d. The potential slip planes were matched with the slip bands detected using secondary electron (SE) imaging in SEM.

The active slip direction was identified using the system with the highest SF. In addition, SEM observations were conducted to study the slip bands after different levels of tensile strain (about 5%, 10%, 15%, and 20%). Each sample was evaluated for over one hundred slip bands in the study; the accuracy of the analysis depended on having statistically significant information. Finally, dislocation motion processes might be linked to the width and number of bands detected.

## 3. Results and Discussion

### 3.1. Mechanical Property

**Figure 3:**
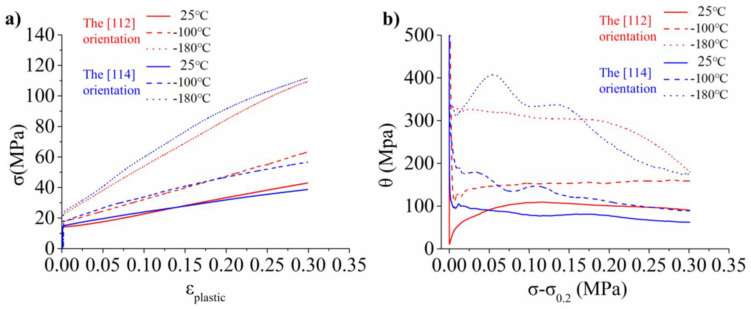
(**a**) Tensile true stress-true plastic strain curves for the single crystal aluminum deformed at different temperatures and (**b**) the corresponding work hardening rate curves.

The true stress–strain curves for the [112] and [114] orientations at the different deformation temperatures are displayed in Figure 3a. It has been discovered that the flow behaviors of the [112] and [114] orientations differ in the −180 °C to 25 °C temperature range. Within the same temperature range, single crystals exhibit similar yield strength regardless of orientation. As expected, the yield strength increases significantly at lower temperatures. The yield strength (σ_0_._2_) of the [112] orientation at 25 °C, 100 °C, and 180 °C were 14.4, 17, and 22.2 MPa, respectively. The yield strength (σ_0_._2_) of the [114] orientation at 25 °C, 100 °C, and 180 °C were 15, 17.7, and 24 MPa, respectively. This is because the main slip systems in [112] and [114] orientations have similar Schmid factors, which are 0.4645 and 0.4745, respectively.

Figure 3b displays the work hardening rate curves for the [112] and [114] orientations. The plastic flow behavior of single-crystal aluminum depends heavily on deformation temperatures and crystallographic orientation. The work hardening rate increases with the decrease in temperature. The deformed material of the deformation substructure is composed entirely of dislocations, making it a “dislocation-mediated plasticity” material [21]. The number and types of active slip systems determine variations in the hardening response. Therefore, the differences between work hardening are explained by observing the evolution of slip bands below.

### 3.2. Microstructure Evolution of Al Single Crystals of the [112] Orientation with Strain

**Figure 4:**
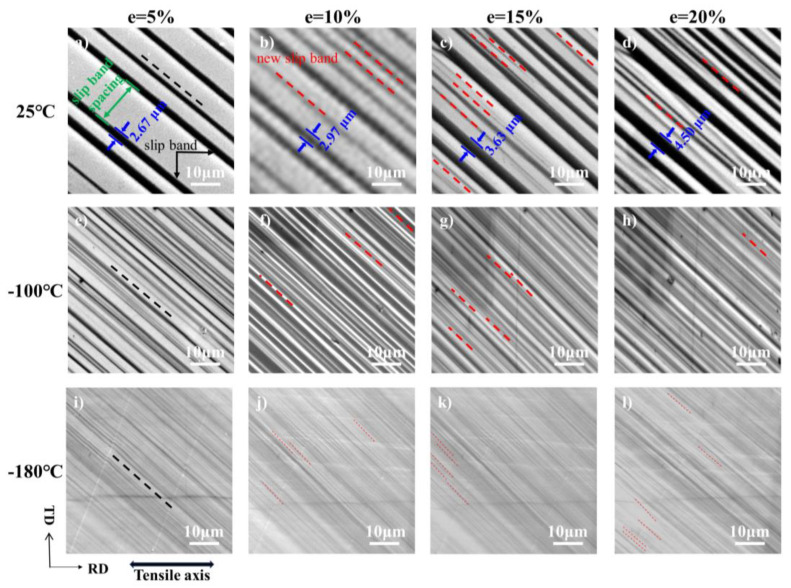
SEM maps showing the evolution of the bands of single crystal aluminum of the [112] orientation with tensile strain up to ~20%: (**a**–**d**), deformed at 25 °C; (**e**–**h**), deformed at −100 °C; (**i**–**l**), deformed at −180 °C. The black dashed lines represent the activation of slip system 11, and the red dashed lines represent newly activated slip bands.

The SEM observations of the development in the bands during the whole uniaxial tensile testing (from e = 5% to e = 20%) at different temperatures are shown in Figure 4. Irrespective of temperature, slip behavior primarily demonstrates a typical “Schmid behavior”, meaning that the slip system is easiest activated with a higher Schmid factor. Slip system 11 is activated at all temperatures. According to Figure 2d, it could be found that the SF of slip system 11 is significantly higher compared with the other slip systems, which was the reason for the single slip behavior to the termination of the test at 25 °C, −100 °C, and −180 °C. In addition, qualitatively, it could be observed that the slip band was coarse at 25 °C (Figure 4a–d) and increased significantly with increasing strain, such as the slip band between the two blue arrows, compared to the low temperature. At −180 °C (Figure 4i–l), the slip bands were diffuse and barely observable. According to the statistics of slip band widths after 5% strain at 25 °C, −100 °C, and −180 °C, the average slip band widths were 1.76, 1.09, and 0.29, respectively. With the increase in strain, new slip bands are constantly generated, as shown by the red dashed lines. Moreover, the density of slip bands was much higher at low temperatures than that at RT under the same strain.

**Figure 5:**
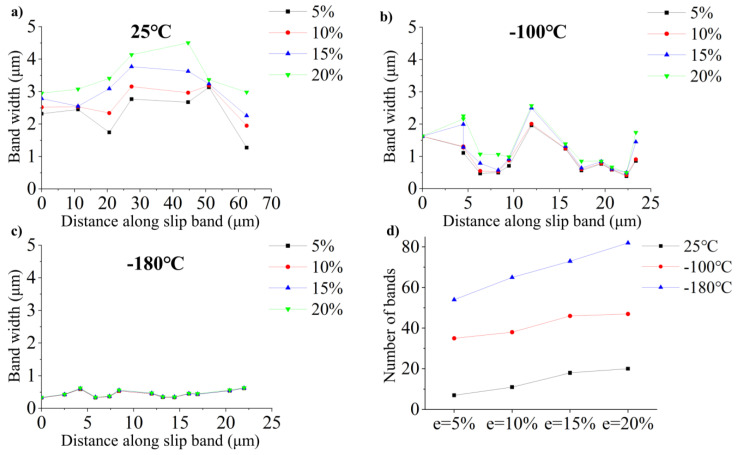
The evolution of the width (**a**–**c**) and number (**d**) of the slip band as measured in Figure 4.

Periodic scans of the same regions were conducted after a 5% strain increment to observe the evolution of individual slip bands. Figure 5a–c show an example where the width perpendicular to the slip bands is plotted as a function of distance for different plastic strains. At 25 °C, it could be observed that, in most of the bands, the width increases as the strain increases, i.e., dislocation intensifies in these slip bands. At −100 °C, only part of the slip band width increased, and the increase was much less than 25 °C. At −180 °C, the slip band width hardly changes, indicating that the slip band had reached saturation before 5% strain.

To assess the band number as a function of plastic strain, the band number is measured as in Figure 4 corresponding to the 25 °C, −100 °C, and −180 °C deformed by tensile testing at different plastic strain levels. The evolution of the number of bands is depicted in Figure 5d. The number of bands increases as temperature decreases throughout the tensile test. The number of bands increased with strain at 25 °C, −100 °C, and −180 °C. But the key difference between 25 °C, −100 °C, and −180 °C was that the number of bands monotonically increased at −180°, respectively, with an initial increase at −100 °C and −25 °C in the number of slip bands followed by saturation. A strong link between this slip band width and the number of bands can be properly rationalized. It is known that the individual plane is softened after the passage of dislocations [22,23]. The nucleation and movement of dislocations along these planes becomes easier than the activation of new slip planes. Therefore, a large number of dislocations preferentially slip along such comparatively softer slip planes. As the temperature decreases, the lattice friction increases, which provides additional resistance to the slip of the dislocation; the softening of the slip plane is severely limited. Therefore, low temperature contributes to the distribution of dislocations along different planes, reducing the width of bands and increasing the number of bands. At the same time, the work hardening of single crystals is also explained, a significant reduction in θ during the initial stage hardening of [112] at 25 °C, typical of stage I behavior of FCC materials, because of mainly single-slip and the generation of stable dislocations [24,25,26]. With the increase in deformation, the work hardening rate is always at a low level due to the softening of the slip plane. At −180 °C, the single crystal exhibited excellent strain-hardening properties for two reasons. First, dislocation mobility is suppressed due to the higher lattice frictional stress at low temperatures. Second, the band’s gradual refinement substructure is driven by deformation [27].

**Figure 6:**
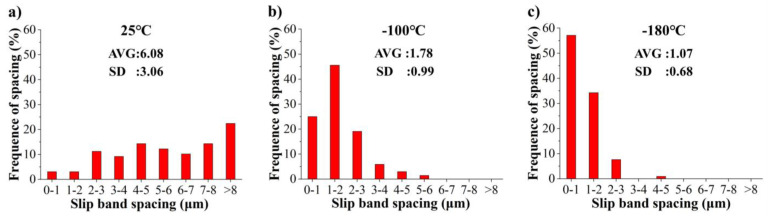
Frequency of slip band spacing of the [112] orientation after a tensile strain of ~5% at (**a**) 25 °C, (**b**) −100 °C, and (**c**) −180 °C.

To quantitatively evaluate the slip band spacing after a tensile strain of 5% at different temperatures, the slip band spacing distribution histograms are summarized in Figure 6a–c. The corresponding average slip band spacing values and their standard deviation are also included next to each slip band spacing distribution. The number of slip bands spacing analyzed at 25 °C, −100 °C, and −180 °C were, respectively, 98, 102, and 128. At 25 °C, −100 °C, and −180 °C, the average slip band spacing was 6.08, 1.78, and 1.07, respectively. With the decrease in temperature, the slip band spacing decreases continuously. The reduction from 25 °C to −100 °C is very large. In addition, the band spacing was uneven at 25 °C. At −180 °C, the band spacing was mostly within 3 μm. At 25 °C, −100 °C and −180 °C, the standard deviations were 3.06,0.99 and0.68, respectively.

**Figure 7:**
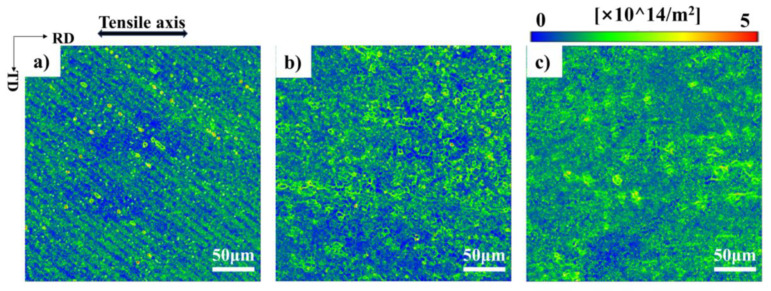
GND diagrams of the [112] orientation after a strain of ~5% deformed at (**a**) 25 °C, (**b**) −100 °C, and (**c**) −180 °C.

As the temperature decreases, improvements are obviously also observed in the uniformity of slip band distribution. The critical stress of activating dislocation slip on specified slip planes is affected by the quantity and distribution of grain boundary [28], precipitates [29], point defects [30], and dislocations tangles [31]. For a single crystal with the [112] orientation, relying on the strain field and the distribution of dislocation [32]. Once reaching a certain stress level, slip system 4 is capable of both nucleating and growing simultaneously. The distribution of dislocation becomes homogeneous as the temperature drops, as shown in Figure 7. At the same time, A more uniform distribution of dislocations at low-temperature deformation has also been reported in other studies. [33,34]. Due to the uneven distribution of dislocation at 25 °C, the critical stress level at which bands can nucleate and grow is different, resulting in the uneven growth region of band nucleation, and the difficulty of slip band growth is also different after nucleation.

**Figure 8:**
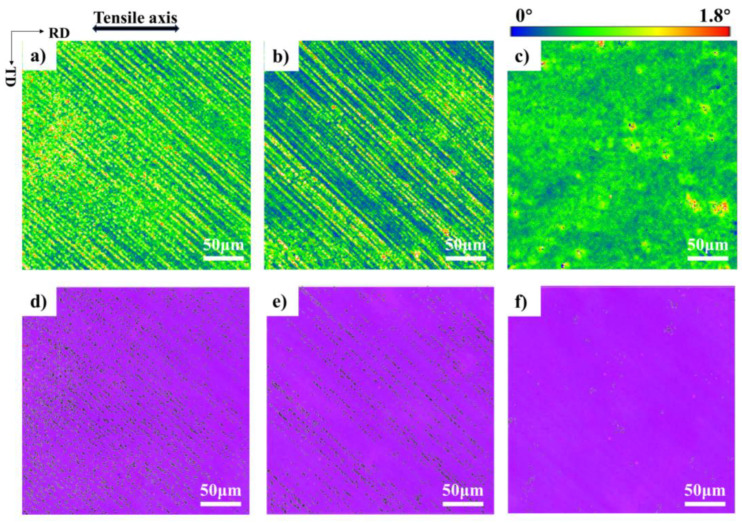
Microstructures of single crystal aluminum of the [112] orientations after tension up to a strain of 15% at (**a**,**d**) 25 °C, (**b**,**e**) −100 °C, and (**c**,**f**) −180 °C. The upper images are KAM diagrams, while the lower images are orientation maps overlaid with low-angle grain boundaries. LAGBs are marked by black lines.

KAM maps are frequently utilized to determine the cracking pattern of materials [35]. The KAM is an indicator of the uniformity of plastic deformation. It helps to measure how evenly the plastic deformation is distributed. Figure 8a–c show the KAM distribution of [112] orientation after a strain of 15% at all temperatures. KAM value decreases gradually from 25 °C to −180 °C, which means that the deformation decreases with the decrease in temperature under the same strain. The green regions indicate the plastic strain localization. At 25 °C, the deformation of the [112] orientation concentrates at the slip bands, suggesting strain localization at the slip bands (Figure 8a). Cracks were more prone to form at these high KAM sites [36]. The coarse slip band will be the potential crack source. At −100 °C, strain localization at the slip bands is alleviated (Figure 8b). At −180 °C, the strain becomes very uniform (Figure 8c). This result can correspond with that of SEM. Because of the higher dislocation density in the coarse slip band at 25 °C, the resulting lattice distortion is greater, and the strain will be localized in the coarse slip band. A large number of fine bands at −180 °C indicates that the slip is dispersed, so the strain is very uniform.

Figure 8d,e show the distribution of low-angle grain boundaries (LAGBs) after a strain of 15% at different temperatures, respectively. The black line denotes the LAGBs, which are defined as 2–10°. To minimize energy, dense dislocations are rearranged into ordered substructures, forming LAGBs [37,38]. It has been observed that the quantity of LAGBs increases in tandem with temperature. There are three reasons for this. First, the cross-slip is promoted, and the dynamic recovery is promoted with the increase in temperature. Dynamic recovery resulted in the organization of dislocations into LAGBs [39]. Second, at 25 °C, a significant number of dislocations are concentrated in the slip band, and dislocation reactions are more likely to occur. Third, higher temperatures can provide more driving energy for dislocation motion. This led to more dislocation reactions. These factors work together to promote the formation of more LAGBs. Studies have demonstrated that the LAGBs can act as a “soft” barrier for movement of dislocation on a larger scale. It permits a portion of dislocations to pass through and obstructs the movement of the remaining dislocations, thereby leading to dislocation multiplication [40]. At 25 °C, the LAGBs in the slip bands lead to dislocation multiplication and further promote the coarsening of the slip bands. The slip band at 25 °C was more prone to the initiation and accumulation of dislocations. Higher strain concentrations due to concentrated dislocations increased the risk of cracking and fracture in the specimen.

**Figure 9:**
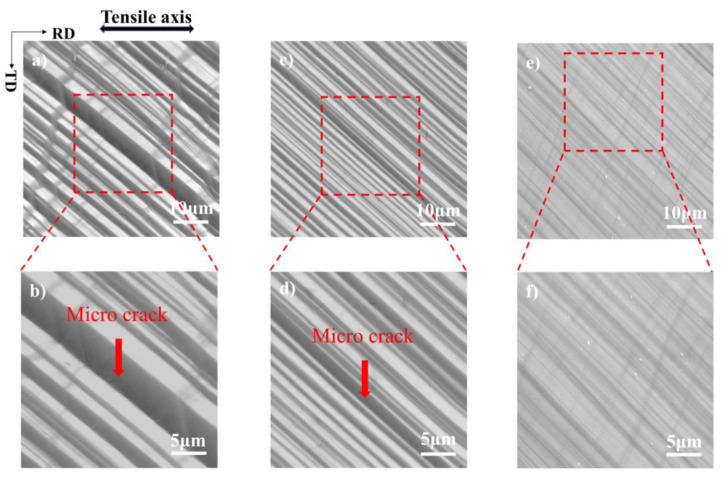
SEM images of single crystal aluminum of the [112] orientations at low and high magnifications after deformation to fracture at (**a**,**b**) 25 °C, (**c**,**d**) −100 °C, and (**e**,**f**) −180 °C.

In summary, at 25 °C, due to the LAGBs to the dislocation movement obstruction and dislocation multiplication mechanism, dislocations accumulated at the slip band. The deformation was uncoordinated due to the perceived local stress concentration at the slip band. The nucleation of micro-cracks along the slip band is caused by inhomogeneous plastic deformation, as shown in Figure 9a,b. At −100 °C, although the slip bands are more dispersed and the density of dislocation in each slip band is less on average, some slip bands still have high dislocation density and LAGBs, and micro-cracks sprout at this part of the slip band, as shown in Figure 9c,d. At −180 °C, the bands are more dispersed, the dislocation density within a single slip band is very low, and the dislocation distribution is more homogeneous, so making the plastic strain very uniform, which improves plasticity. Although the further movement of the dislocation is inhibited by the decrease in temperature and the internal stress is generated when the dislocation stops moving, at low temperatures, the single crystal will release the internal stress by opening more new slip bands, thus avoiding the generation of micro-cracks as depicted in Figure 9e,f.

### 3.3. Slip Band Evolution of Al Single Crystals of the [114] Orientation

**Figure 10:**
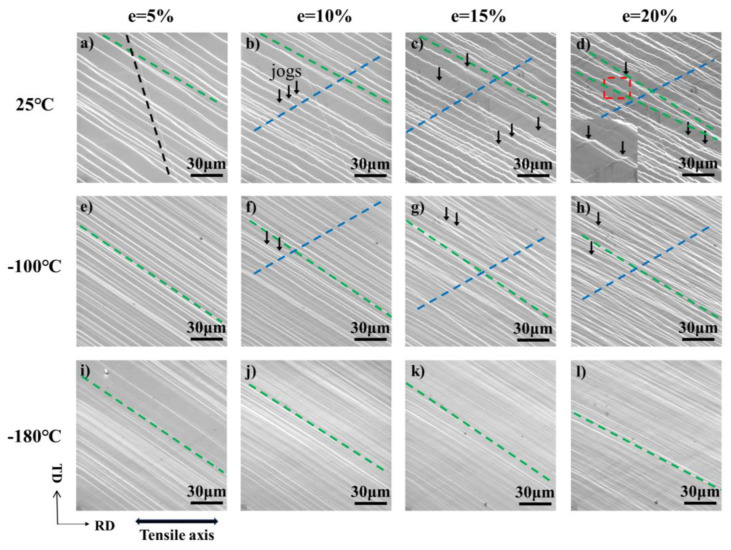
SEM maps showing the evolution of the slip bands of single crystal aluminum of the [114] orientation with tensile strain up to ~20%: (**a**–**d**), deformed at 25 °C; (**e**–**h**), deformed at −100 °C; (**i**–**l**), deformed at −180 °C. Slip system 4, slip system 7, and slip system 12 are marked with blue, green, and black dashed lines, respectively.

**Figure 11:**
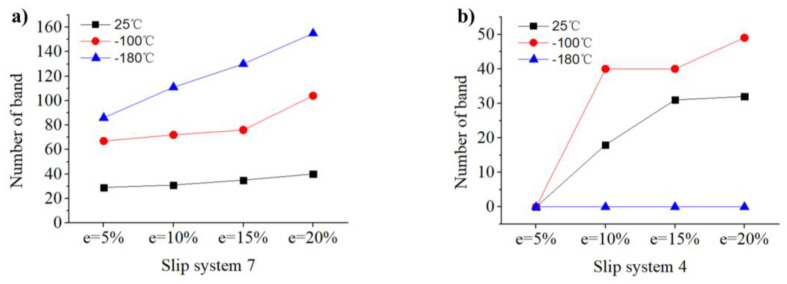
The evolution of the number of the band as measured in Figure 10: (**a**) slip system 7 and (**b**) slip system 4.

During the whole tensile testing (from e = 5% to e = 20%) at 25 °C, −100 °C, and −180 °C of [114] orientation, the evolution of the bands is shown in Figure 10, the slip bands shown by green and blue dotted line corresponds to the (111) [0–11] and (−111) [0–11] slip system, respectively, namely slip system 7 and slip system 4. The Schmid factors of slip system 7 and 4 were 0.4745 and 0.4248, respectively. To assess the evolution of bands as a function of plastic strain, a lot of bands in different slip systems were measured from the SEM maps in Figure 10. The evolution of band number is depicted in Figure 11a,b, corresponding to slip systems 7 and 4. At 25 °C, slip systems 7 and 12 are activated at the early stage of strain (since the number of activations of slip system 12 is very small and does not increase, the capacity to accommodate the strain is very limited, and this paper will not discuss it.), as indicated by Figure 10a’s green dotted line, the slip trace of slip system 7 is always changing due to the constraint of uniaxial stretching [41]. Because the Schmid factor of slip system 7 is larger than that of slip system 4, slip system 4 is activated after 10% strain, as indicated by Figure 10b’s blue dotted line. In the meantime, slip system 4 keeps multiplying until the 15% strain approaches saturation. The slip system 7 increases slowly during the whole tensile testing. At −100 °C, slip system 7 is activated first, but there are more bands at the initial stage of strain than at 25 °C. This may be due to the yield strength increasing as temperature decreases, and greater stress is sufficient to activate more slip planes. Slip system 4 is also activated at 10% strain. However, compared with deformation at 25 °C, the growth rate of slip system 4 is faster. Due to the greater number of activations of slip system 7, more dislocation sources are provided for slip system 4. Upon reduction of the temperature to −180 °C, distinct mechanisms were observed in the SEM images, as illustrated in Figure 10i–l Firstly, the slip bands changed from multi-slip at 25 °C to single-slip, and only slip system 7 was activated. Three reasons contribute to this. First, the decrease in temperature increased the lattice resistance, making it harder for slip systems to initiate. [42]. Second, although a large number of slip system 7 are activated in the early deformation, which can provide a higher amount of possible dislocation sources for slip system 4, it also obstructs slip system 4. Third, the activation of the new slip band means that more strain can be accommodated. According to Figure 11a, the growth rate of slip system 7 at −180 °C is much higher than that at −100 °C and 25 °C, and the increase in the number of slip system 7 is sufficient to accommodate more strain without the need to activate the slip system 4 to coordinate the deformation.

In addition, from Figure 10b–d,g,h, it can be observed that the bands showed a zigzag shape at 25 °Cand −100 °C after 10% strain. The black arrow indicates the locations where jogs can be seen at the intersection of bands. Due to the movement of dislocation at (−111) plane toward outside the material surface, dislocation at (111) plane was cut along the moving direction of dislocation at (−111) plane, resulting in the formation of a jog between the original dislocation line and the dragged dislocation line [43]. Due to a large number of slip steps of dislocations that can form a slip band, the jogs in Figure 10 consist of multiple individual jogs. Compared with 25 °C, the number of slip bands is more after the same stain at −100 °C, and the number of dislocations passing through a single slip band is less, so the jog generated at 25 °C is more obvious than that at −100 °C. And as the strain increases, more and more dislocations are reacted, so the jog becomes more and more obvious.

**Figure 12:**
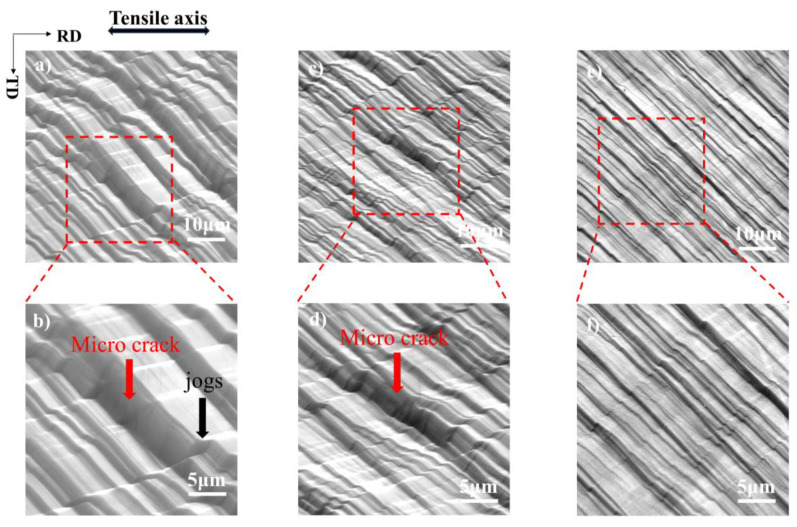
SEM images of single crystal aluminum with the [114] orientations at low and high magnifications after deformation to fracture at (**a**,**b**) 25 °C, (**c**,**d**) −100 °C, and (**e**,**f**) −180 °C.

Dislocation jogs severely influence the dislocation slip. After the jog is formed, the movement of the dislocation in this direction is blocked, resulting in dislocation pile-up. Stroh et al. [44] demonstrated that dislocation pile-up causes high strain concentration, which may lead to cleavage cracks. Figure 12a is an example; at 25 °C, multiple slip systems are activated after a certain level of plastic deformation, and a clear jog, marked by a black arrow, is visible. Figure 12b provides a higher magnification of the jog. Due to numerous dislocation line jogs at the intersection of multiple slip systems, high localization of strain near the jog induces obvious surface roughness and crack nucleation (marked by red arrow).

In contrast, after some level of deformation at −100 °C, there is also a certain degree of strain localization near the jog, as shown in Figure 12c,d. However, since the band is more dispersed at −100 °C, there are fewer dislocations within a single slip band, and the dislocation pile-up at the jogs is reduced because the increase in lattice friction provides additional lattice resistance to the dislocations’ motion. The degree of stress concentration and deformation localization is lighter than at 25 °C. At −180 °C, the slip is diffuse, numerous fine bands are observed, and the second slip system is activated after a large deformation. The jog degree is very light, promoting a more uniform dislocation distribution, and the deformation is very uniform, as illustrated in Figure 12e,f, which significantly improves plasticity.

**Figure 13:**
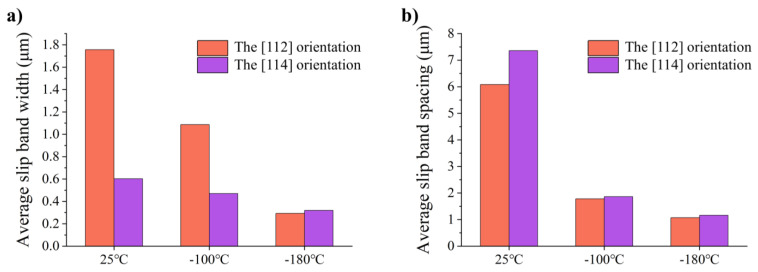
(**a**) Average slip band widths and (**b**) average slip band spacings after tension up to a strain of 5% at different temperatures in single crystal aluminum with the [112] and [114] orientations.

As shown in Figure 13, both the average slip band widths and spacings decrease with the decrease in temperature. The specific values are shown in Table 1. At 25 °C, the individual plane is softened after the passage of dislocations. It is easier for dislocations to nucleate and slip in this slip plane than to activate a new slip plane, and the high mean free path increases the distance between the dislocation source and the dislocation pile-up, thus making the back stress of the dislocation source lower, so that the dislocation source can produce more dislocations before the back stress of the dislocation pile-up reaches a threshold [45], resulting in localization of slip. These coarse slip bands form crack sources under high strain, which adversely affects plasticity. The decrease in temperature leads to the increase in lattice friction stress and the decrease in cross-slip activity [13,14]. The mean free path of the dislocation decreases, so the dislocation source is inhibited faster. At this point, the band might be considered completely developed [27]. Then, another dislocation source is activated. More dislocation sources will be generated under the same strain at low temperatures, and the number of dislocation sources is inversely proportional to the slip band spacing [45]. Therefore, the slip band spacing is smaller at low temperatures. The density of the slip plane increases continuously during the whole deformation process, resulting in dynamic slip band refinement, making the deformation more uniform and inhibiting the generation of cracks. Moreover, dynamic slip band refinement enables the specimen to maintain excellent work hardening during the whole deformation process until the slip band spacing reaches a critical value [27]. However, because the [114] orientation at 25 °C and −100 °C is multi-slip, the activation of multi-slip disperses the dislocations so that the number of dislocations passing through a single plane is less than that of the [112] orientation. Therefore, the average slip band widths of the [114] orientation are smaller than that of the [112] orientation at 25 °C and −100 °C. However, dislocation slip at 25 °C and −100 °C is still local compared to −180 °C. Therefore, when the dislocations in the two slip systems are jogged, it causes serious dislocation pile-up and leads to the generation of cracks, reducing the plasticity. For the [114] orientation, the dynamic slip band refinement at −180 °C can also significantly improve the strength and plasticity. It is worth noting that at −180 °C, both orientations are single slips, so the average slip band width and spacing are very similar. The consistency of slip behavior of the two orientations at −180 °C weakens the orientation effect and eventually improves the deformation uniformity of the polycrystals at low temperature A forthcoming publication will center around those works, demonstrating that the anisotropy of orientation was indeed well weakened.

In summary, at RT, due to the orientation effect, the two orientations exhibit different slip modes, which results in distinct work hardening. Although the causes of dislocation accumulation vary, they all lead to uneven dislocation distribution and deformation, which are detrimental to plasticity. At −180 °C, they all transition into a dense single system slip mode, which gives the sample a better work-hardening ability and makes the dislocation distribution more uniform, avoiding crack nucleation. The slip plane is continuously activated with the strain so that the sample can accommodate more deformation, which improves the plasticity. However, the study in this paper cannot involve the influence of grain boundaries. Grain boundaries usually play multiple roles, such as dislocation source and barrier, which have an important influence on dislocation behavior and plastic deformation. Therefore, the low-temperature deformation mechanism of polycrystalline aluminum will be studied in detail in the future.

## 4. Summary and Conclusions

The objective of this work was to investigate the relation between temperature and mechanical response by studying the deformation mechanisms in aluminum. With that goal, the single crystal samples with the [112] and [114] orientations were tested at 25 °C, −100 °C, and −180 °C. EBSD-assisted slip trace analysis was utilized to study the evolution of slip bands. Primary findings and conclusions were summarized as follows:

1. The slip band width and spacing declined with the decrease in temperature, irrespective of the orientation. At −180 °C, high slip-plane density was found in the [112] and [114] orientations, and the samples showed excellent strain-hardening properties and a more homogeneous deformation during tensile tests.
2. At room temperature, due to the slip plane’s softening and the dislocation multiplication mechanism, the slip bands became coarse with the increase in strain. When the temperature was decreased to −180 °C, dynamic slip band refinement occurred, which prevented slip localization and inhibited the generation of cracks.
3. The slip mode was influenced by orientation and temperature. Single slip was the main slip mode of the [112] orientation at all the temperatures investigated. The slip behavior of the [114] orientation changed from multi slip at RT to single slip at −180 °C, which avoided the dislocation pile-up near the jogs and inhibited the generation of cracks.

This study contributes to a better understanding of the microscopic mechanisms that cause the simultaneous increase in strength and ductility of aluminum at cryogenic temperatures.

## Figures and Tables

**Table 1:** Average slip band widths and spacings after tension up to a strain of 5% at different temperatures in single crystal aluminum of the [112] and [114] orientations.

	Average Slip Band Widths (μm)		Average Slip Band Spacings (μm)	
	[112]	[114]	[112]	[114]
25 °C	1.76	0.60	6.08	7.36
−100 °C	1.09	0.47	1.78	1.86
−180 °C	0.29	0.32	1.07	1.16

## Data Availability

The data that support the findings of this study are available from the corresponding author upon reasonable request.
